# Differences in physical symptoms between those with and without kidney disease: a comparative study across disease stages in a UK population

**DOI:** 10.1186/s12882-021-02355-5

**Published:** 2021-04-22

**Authors:** Thomas J. Wilkinson, Daniel G. D. Nixon, Jared Palmer, Courtney J. Lightfoot, Alice C. Smith

**Affiliations:** 1 Leicester Kidney Lifestyle Team, Department of Health Sciences, University of Leicester, Leicester, UK; 2 Leicester NIHR Biomedical Research Centre, Leicester, UK

**Keywords:** Kidney diseases, Diagnosis, Symptoms, Transplantation, Dialysis, Fatigue, Pain

## Abstract

**Background:**

Those living with kidney disease (KD) report extensive symptom burden. However, research into how symptoms change across stages is limited. The aims of this study were to 1) describe symptom burden across disease trajectory, and 2) to explore whether symptom burden is unique to KD when compared to a non-KD population.

**Methods:**

Participants aged > 18 years with a known diagnosis of KD (including haemodialysis (HD) and peritoneal dialysis (PD)) and with a kidney transplant) completed the Leicester Kidney Symptom Questionnaire (KSQ). A non-KD group was recruited as a comparative group. Multinominal logistic regression modelling was used to test the difference in likelihood of those with KD reporting each symptom.

**Results:**

In total, 2279 participants were included in the final analysis (age 56.0 (17.8) years, 48% male). The main findings can be summarised as: 1) the number of symptoms increases as KD severity progresses; 2) those with early stage KD have a comparable number of symptoms to those without KD; 3) apart from those receiving PD, the most frequently reported symptom across every other group, including the non-KD group, was ‘feeling tired’; and 4) being female independently increased the likelihood of reporting more symptoms.

**Conclusions:**

Our findings have important implications for patients with KD. We have shown that high symptom burden is prevalent across the spectrum of disease, and present novel data on symptoms experienced in those without KD. Symptoms requiring the most immediate attention given their high prevalence may include pain and fatigue.

**Trial registration:**

The study was registered prospectively as ISRCTN11596292.

**Supplementary Information:**

The online version contains supplementary material available at 10.1186/s12882-021-02355-5.

## Introduction

Kidney disease (KD) is a global health problem affecting between 8 and 12% of the worldwide population. As kidney function declines, morbidity and mortality increases. In the advanced stages of KD, appropriate renal replacement therapy (either dialysis or transplantation) is needed to sustain life [1]. It is now well-established that those living with kidney dysfunction report extensive symptom burden, and there is a growing body of evidence to suggest that symptom burden is an important predictor of reduced health related quality of life (HRQoL) [2–8]. Common symptoms experienced by many individuals living with KD include fatigue, pain, pruritus, irritability, anxiety, and nausea [3, 9–11].

The assessment of symptom burden in all KD patients is important in clinical management. However, evidence shows that healthcare providers frequently under-recognize and under-treat the physical symptoms of KD [12]. Understanding the degree to which symptoms affect this large, and growing, population may help facilitate the implementation of symptom-alleviating therapies that may in turn favourably impact HRQoL [2]. Although the literature has generally focused on the prevalence of symptoms among those treated by dialysis, more evidence has started to emerge showing that symptom burden is also evident in those with earlier stages of KD [9]. However, research to-date into non-dialysis cohorts has almost exclusively been conducted in those with Stage 4 or higher KD [3] and, in particular, evidence in Stages 1–2 KD is lacking. Such data may provide important information into the development of symptoms in those with earlier KD.

The exact nature of how symptoms change across stages is somewhat inconsistent, likely due to the heterogeneous and small sampled populations studied, and the assorted methods of symptom assessment and questionnaires. In many individuals, KD exists as part of a multimorbid array of different health conditions [13] which makes the identification and designation of symptoms to KD itself difficult. Whilst one may argue that symptoms should be treated on presentation regardless of health status, understanding how the presence of KD modulates symptom burden is vital. To our knowledge, no research has investigated whether or not the symptoms experienced by those living with KD are unique to the condition; or are simply a function of other health-related or demographic characteristics (e.g., age, sex, comorbidity) independent of KD itself. The aims of this study were two-fold: 1) to describe symptom burden across the disease trajectory, and 2) to explore whether symptom burden is unique to KD when compared to a non-KD population.

## Methods

### Study design

This is an analysis of questionnaire data returned as part of the ‘Intramuscular and Inflammatory Response to Acute Exercise in Chronic Disease (I-RACE)’ study conducted between November 2017 and July 2020. Participants were recruited and provided (either in person during scheduled appointments or treatments, or via the post) a survey pack containing a series of questionnaires. All participants with KD were recruited from seven sites across the UK. Participants *without* KD (to their knowledge) were opportunistically recruited through contacts of research sites, or via local university and hospital sites. All KD participants completed the survey independently, either at home or during their appointment/treatment, and returned it to the research team who then extracted clinical details from electronic medical records. The study was registered prospectively as ISRCTN11596292. National ethical approval was granted by the East Midlands - Derby Research Ethics Committee (ref: 17/EM/0357). All KD participants provided written informed consent. In those without KD, an information sheet detailing the study was provided and informed consent was implied upon the return of the questionnaires. As such, no identifiable information was collected for the non-KD group. The study was conducted in accordance with the Declaration of Helsinki.

### Study population

Participants aged ≥18 years with a known diagnosis (from medical records) of KD (including those on dialysis, both haemodialysis (HD) and peritoneal dialysis (PD)) and with a kidney transplant) were included. Estimated glomerular filtration rate (eGFR, shown as ml/min/1.73^2^) calculated using the EPI formula and patients were stratified into appropriate non-dialysis stages (Stage 1–2: ≥60; Stage 3: 30–59: Stage 4: 15–29). Those with an eGFR < 15 but not requiring dialysis were defined as Stage 5ND. A pragmatic non-KD group was opportunistically recruited through hospital and university staff across all sites. Participants were asked to also hand surveys out to appropriate family and friends. The non-KD group provided anonymous responses via the return of the questionnaire. No personal identifiable information was collected for this group.

### Leicester kidney symptom questionnaire (KSQ)

The Leicester Kidney Symptom Questionnaire (KSQ) was used for symptom assessment. The KSQ has been validated for use in those with non-dialysis KD [14] and is being established as a measure of symptom burden in those with KD [15–17]. The KSQ assesses the frequency (how often a patient experienced a symptom) of 13 kidney-related symptoms (itching; sleep disturbance/insomnia; loss of appetite; feeling tired; pain in bones/joints; poor concentration/mental alertness; loss of libido; loss of muscle strength/power; shortness of breath; cramp/muscle stiffness; restless legs; the need to urinate more often (night and/or day); feeling cold) on a 5-point Likert scale. Frequency was assessed using the following response options: (0) ‘Never’, (1) ‘Less than once a week’, (2) ‘1–2 times a week’, (3) ‘Several times a week’ and (4) ‘Every day’. Symptoms were classified as binary (present or absent). A symptom was defined as being present if experienced at least once a week or more; absence of symptom was defined if a participant responded by either experiencing the symptom ‘never’ or ‘less than once a week’.

### Statistical analysis

**Fig. 1:**
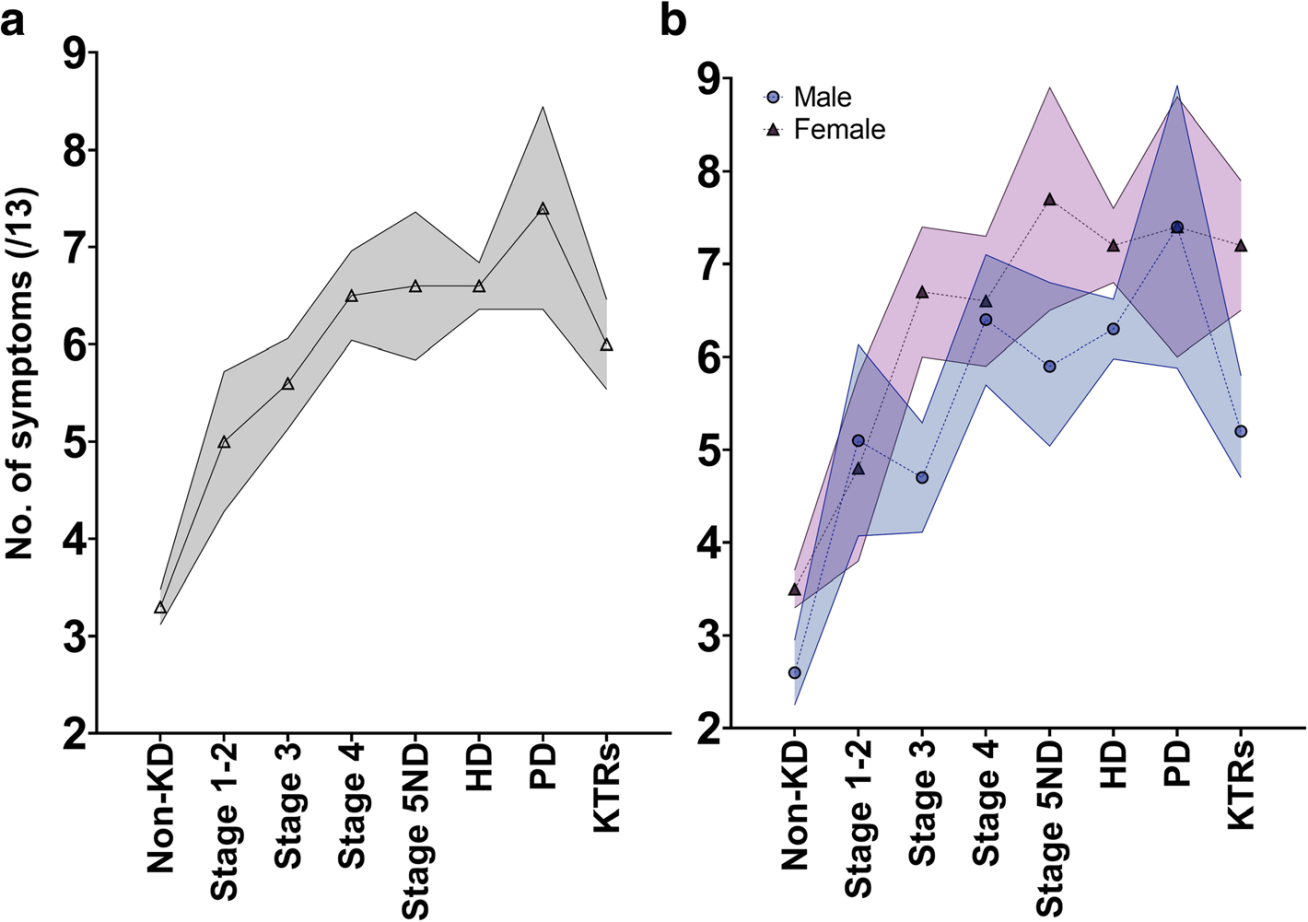
The mean number of symptoms across disease stages in total **a** and stratified by sex **b**. Left **a** shows mean number of symptoms across groups; Right **b** shows number of symptoms stratified by sex and group. Data shown as mean and 95% confidence interval (coloured shaded area). KD = Kidney disease; ND = Non-dialysis; HD = Haemodialysis; PD = Peritoneal dialysis; KTRs = Kidney transplant recipients. Data used to generate figure can be found in Supplementary Material 2

Data was analysed using IBM SPSS Statistics Version 26. Unless otherwise stated, continuous data presented as mean and standard deviation. For Fig. 1, the 95% confidence interval (95%CI) was estimated. Percentages shown as valid percent (i.e., excluding missing data). Number of missing symptom data is shown in Supplementary Material 1. Analysis of the differences in the number of symptoms between non-KD and the KD groups was tested using univariate general linear models. Differences in responses was assessed using Chi-Square testing (with Bonferroni correction). Multinominal logistic regression modelling (adjusted for age, sex, ethnicity, and no. of comorbidities) was used to test the difference in likelihood of those with KD reporting each symptom. Data shown as Odds Ratio (OR) with 95%CI. An OR greater than 1 indicated an increased likelihood of having this symptom. The following comorbidities were self-reported as part of the survey and used in the analysis (diabetes, cancer, heart disease, stroke, peripheral vascular disease, asthma/chronic pulmonary obstructive disease, liver problems, musculoskeletal conditions, neurological, mental health illness, and other (free text box for participants to input other conditions). Hypertension was extracted from medical records of patient participants. As an exploratory observational study, no formal sample size was calculated, and we aimed to recruit as many participants as possible during the study period. Statistical significance was recognised as *P* < .050.

## Results

### Clinical and demographic characteristics

In total, 2425 participants were recruited into the study. Sixty-seven patients without a KD classification/stage (*n* = 18) or eGFR (*n* = 49) measurement were excluded as they were unable to be classified with a KD staging. Seventy-four participants did not complete the KSQ and were also excluded. Those who did not return the KSQ were older (mean age, 61.3 (13.4) vs 56.0 (17.8) years (*P* = .012)). No significant difference in ethnicity (*P* = .617) or sex (*P* = .566) was seen in those who returned the KSQ compared to those who did not. A greater proportion of KTRs (11.7%) did not return the KSQ than other groups (*P* < .001). Those who returned the KSQ had significantly less comorbidities (P < .001). As such, 2279 participants were included in the final analysis.

Table 1 shows the clinical and demographic characteristics of the 2279 participants included, stratified by stages. In summary, the mean age of all participants included was 56.0 (17.8) years and made up of 48% male. The majority of participants were White British (74%). The cohort included 853 non-KD participants (37%) whilst 530 were non-dialysis (ND) Stage 1–5, 630 were receiving HD (28%) and 230 (10%) had received a transplant (KTRs).

**Table 1:** Participant demographics and clinical characteristics

	Non-KD reference	Non-dialysis kidney disease				HD	PD	KTRs
		Stage 1–2	Stage 3	Stage 4	Stage 5ND			
	*N = 853*	*N = 72*	*N = 182*	*N = 194*	*N = 82*	*N = 630*	*N = 28*	*N = 230*
Age (years)	46.6 (16.1)	46.0 (16.2)	64.2 (14.9)	70.7 (15.1)	66.0 (18.0)	63.0 (14.6)	55.1 (18.2)	51.8 (13.5)
Sex (male *n*, %)	232 (27%)	36 (50%)	99 (54%)	105 (54%)	57 (63%)	395 (63%)	17 (61%)	143 (62%)
**Ethnicity**								
*White British (n, %)*	607 (72%)	55 (78%)	162 (90%)	175 (91%)	73 (81%)	391 (63%)	17 (63%)	181 (79%)
*South Asian (n, %)*	96 (11%)	8 (11%)	7 (4%)	12 (6%)	7 (8%)	91 (15%)	6 (22%)	24 (11%)
*Other (n, %)*	137 (16%)	8 (11%)	12 (7%)	6 (3%)	10 (11%)	140 (23%)	4 (15%)	24 (11%)
eGFR (ml/min/1.73m^2^)	a	77.6 (11.7)	41.0 (7.9)	22.7 (4.1)	11.8 (2.8)	–	–	46.8 (22.0)
CRP (mg/L)	a	10.4 (23.5)	11.0 (19.4)	18.4 (56.1)	11.6 (16.0)	18.9 (34.5)	22.3 (39.5)	13.9 (32.4)
Haemoglobin (g/dL)	a	136.8 (157.5)	88.0 (55.9)	93.8 (47.6)	98.8 (34.6)	103.4 (40.5)	101.8 (21.9)	100.5 (48.2)
Albumin (g/dL)	a	41.7 (4.1)	41.0 (6.3)	40.9 (4.5)	40.1 (5.6)	38.6 (5.0)	36.2 (5.7)	42.1 (5.9)
HbA1C (%)	a	5.7 (0.9)	6.9 (1.6)	6.8 (1.5)	6.8 (1.9)	6.5 (1.6)	6.8 (1.6)	6.0 (1.3)
Dialysis vintage (mths)	a	b	b	b	b	40.8 (46.0)	12.3 (20.7)	–
Transplant vintage (mths)	a	b	b	b	b	b	b	67.5 (80.6)
Kt/V (ratio)	a	b	b	b	b	1.81 (5.85)	a	b
URR (%)	a	b	b	b	b	99.5 (451.3)	a	b
No. of comorbidities	1.2 (1.4)	1.6 (1.7)	3.5 (2.8)	4.6 (2.6)	4.2 (2.0)	2.5 (1.7)	3.2 (1.9)	2.7 (2.1)
*Hypertension (n, %)*	a	27 (28%)	121 (67%)	144 (74%)	63 (70%)	409 (65%)	20 (71%)	141 (61%)
*Other CVD (n, %)*	66 (8%)	7 (10%)	46 (25%)	73 (38%)	29 (35%)	243 (39%)	5 (18%)	29 (13%)
*Diabetes (n, %)*	35 (4%)	6 (8%)	47 (26%)	81 (41%)	36 (40%)	261 (41%)	12 (43%)	53 (23%)
*MSK problems (n, %)*	233 (27%)	7 (10%)	44 (24%)	55 (28%)	20 (22%)	70 (11%)	11 (39%)	45 (20%)
*Respiratory conditions (n, %)*	109 (13%)	6 (8%)	23 (13%)	27 (14%)	10 (11%)	64 (10%)	2 (7%)	15 (7%)

Data shown as mean and standard deviation, unless otherwise stated. Frequencies reported as number and valid percentage*. KD* Kidney disease*, ND* Non-dialysis*, HD* Haemodialysis*, PD* Peritoneal dialysis*, KTRs* Kidney transplant recipients*, eGFR* Estimated glomerular filtration rate*, CRP* C-reactive protein*, URR* Urea reduction ratio*.* ^a^ = not measured; ^b^ = not applicable to group. Other CVD includes heart disease and stroke; respiratory conditions include chronic obstructive pulmonary disorder and asthma

### Number and frequency of symptoms reported across groups

The number of symptoms (out of a total of 13) for each group can be found in Fig. 1. The number of symptoms increased as disease stages progressed, before alleviating in KTRs. Those without KD (mean number of symptoms 3.3, 95%CI 3.1 to 3.5) and those in Stage 1–2 (mean 5.0, 95%CI 4.3 to 5.7) had a significantly lower number of symptoms (than all other groups (non-KD: *P* < .001; Stage 1–2: *P* < .001 to .037), adjusted for age, sex, and ethnicity). There was no statistically significant difference in the number of symptoms between those in Stage 4 (6.5, 95%CI 6.0 to 7.0)), 5ND (6.6, 95%CI 5.8 to 7.4), PD (7.4, 95%CI 6.4 to 8.4) or HD (6.6, 95%CI 6.4 to 6.8). There was no difference in the number of symptoms between those in Stage 3 (5.6, 95%CI 5.1 to 6.1) and KTRs (6.0, 95%CI 5.5 to 6.5) (*P* < .355).

**Fig. 2:**
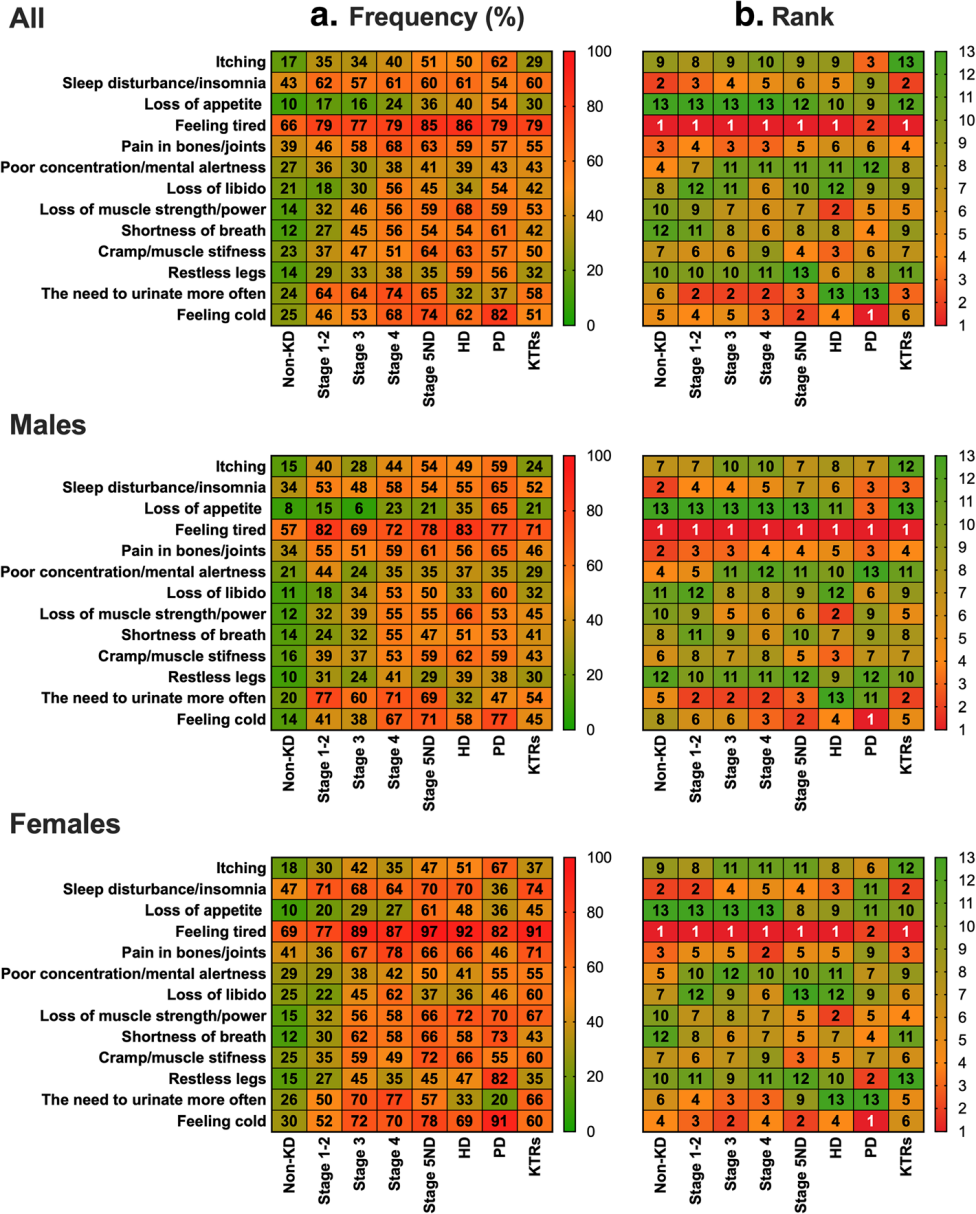
Heatmaps showing the frequency **a** and rank **b** of symptoms across disease stages and sexes. Left heatmap shows the frequency of symptoms reported across each stage as a percentage; Right heatmap shows each symptom ranked based on its frequency (e.g., 1 = most frequently reported symptom, 13 = least frequently reported symptom). Percentages shown as valid percent (i.e. excluding missing data). Present symptom defined as being present at least once a week or more; absence of symptom was defined as either never or < 1 week. KD = Kidney disease; ND = Non-dialysis; HD = Haemodialysis; PD = Peritoneal dialysis; KTRs = Kidney transplant recipients. Data used to generate figure can be found in Supplementary Material 2

**Fig. 3:**
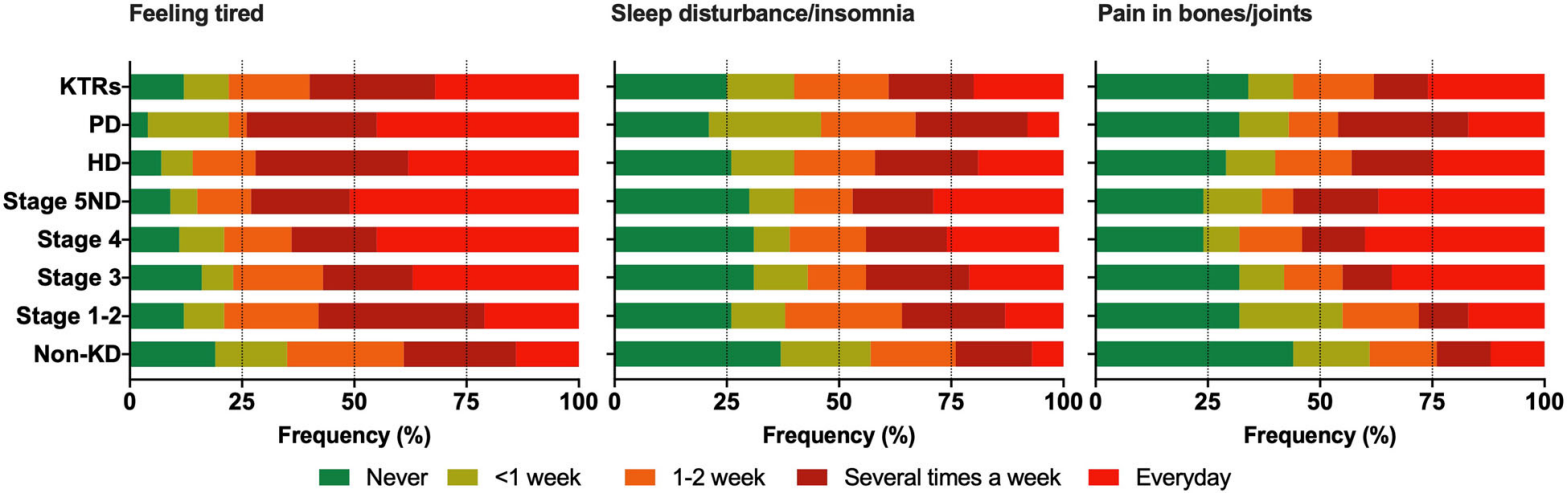
Breakdown of responses in top three symptoms across groups. Data shown as proportion of responses in the top three symptoms reported in all participants. ND = Non-dialysis; HD = Haemodialysis; PD = Peritoneal dialysis; KTRs = Kidney transplant recipients. Data used to generate figure can be found in Supplementary Material 3

Figure 2 shows the frequency and corresponding rank of each symptom across each disease stage, and also stratified by sex. Apart from feeling tired (66%), all the other symptoms were found in < 50% of respondents. Overall, the frequency of all symptoms generally increased as disease stages worsened. Those with a transplant (KTRs) had lower symptom burden than those with advanced non-dialysis stages. Apart from those receiving PD, the most frequently reported symptom across every other group, including the non-KD group, was ‘feeling tired’ with between 66 and 86% of all participants reporting it as a symptom at least once a week or more. The most commonly reported symptom in PD was ‘feeling cold’ (82%), with ‘feeling tired’ ranked second (79%). A full breakdown of frequency responses for the top three symptoms (feeling tired, reported in 76% of all participants; sleep disturbance/insomnia, 54%; pain in bones/joints, 52%) reported across all participants can be found in Supplementary Material 3. Compared to the non-KD group, the proportion of patients experiencing these three symptoms ‘everyday’ was significantly higher in Stages 3, 4, 5ND, HD, and KTRs (Fig. 3).

The need to urinate more often was the second most common symptom in those with Stage 1–2, 3, and 4, and it was the third was common symptom in those in Stage 5ND and in KTRs. The second was common symptom in those on HD was loss of muscle strength/power (68%), whilst sleep disturbance/insomnia was the second most frequent symptom in 60% of KTRs. Pain in bones/joints was the third most frequently reported symptom in those without KD but also in those in Stage 3 and 4. The least reported symptom was loss of appetite in the non-KD, Stage 1–2, 3, and 4 groups.

### Likelihood of reporting symptoms across KD stages compared to non-KD group

**Fig. 4:**
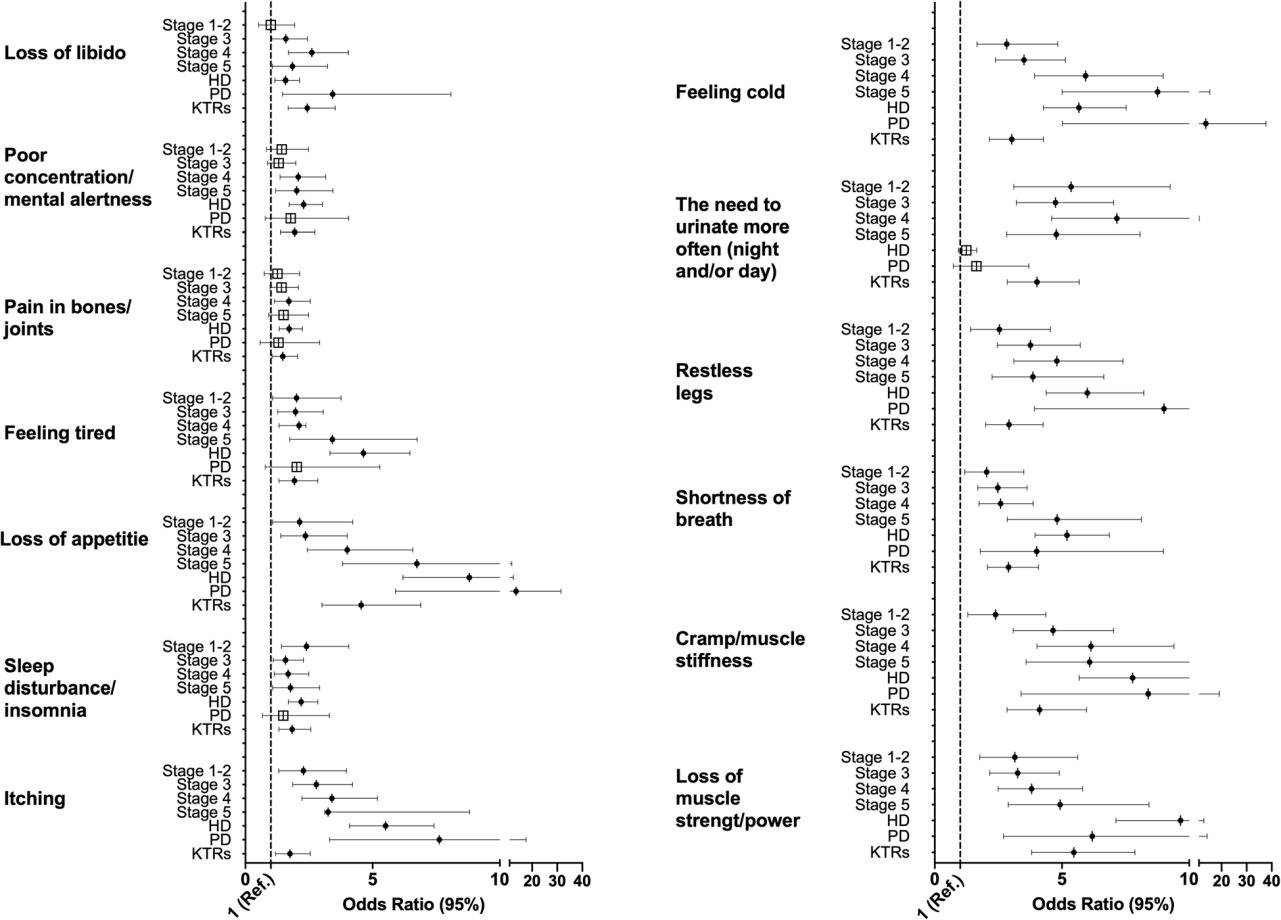
Odds Ratio of having a symptom present across groups. Data shown as Odds Ratio with 95% confidence intervals. The dotted line represents an Odds Ratio of 1 (non-kidney disease reference group). An Odds Ratio greater than 1 indicated an increased likelihood of having this symptom. Closed black dots indicate a significant Odds Ratio; larger open square indicates no statistically significant difference to reference group. A present symptom was defined as being present at least once a week or more; absence of symptom was defined as either never or < 1 week. For males, loss of libido also included presence of erectile dysfunction. Tested using multinominal logistic regression modelling (adjusted for age, sex, ethnicity, and no. of comorbidities). ND = Non-dialysis; HD = Haemodialysis; PD = Peritoneal dialysis; KTRs = Kidney transplant recipients. Data used to generate figure can be found in Supplementary Material 4

Figure 4 shows the likelihood of reporting symptoms across KD stages compared to non-KD group. Patients across all stages had a significantly increased likelihood of itching compared to the non-KD reference group. Those with Stage 5ND (OR = 5.25), HD (OR = 5.51), and PD (OR = 7.62) were 5 to 8 times more likely to report itching as a symptom. Those with KD were approximately twice (OR = 1.58 to 2.40) as likely to report ‘sleep disturbance/insomnia’ as a symptom. The likelihood of loss of appetite being reported as a symptom increased with disease progression; those on HD and PD were 8 (OR = 8.80) to 13 (OR = 13.60) times more likely, respectively, to experience this symptom than the non-KD reference group. Feeling tired was two to five times as likely to be reported in those with KD. The odds of reporting pain in bones/joints were increased in those with Stage 4 (OR = 1.71), HD (OR = 1.72), and KTRs (OR = 1.45) only. Poor concentration was more likely in those with advancing disease (Stage 4, OR = 2.08; Stage 5ND, OR = 2.02; HD, OR = 2.29; KTRs, OR = 1.94).

Loss of libido was increasingly likely in those with KD, although not in those with Stage 1–2 (OR = 1.00). Loss of muscle strength/power was approximately 3 to 10 times more likely to be reported in those with KD; those on HD had a 10x increased odds (OR = 9.66) of reporting this as a symptom. The odds of having shortness of breath increased with KD progression and was significantly more likely to be reported in all stages (OR = 2.39 to 8.39). Cramp/muscle stiffness was twice as likely to reported in those with KD, with odds increasing in those with advanced disease (Stage 5ND, OR = 4.81; HD, OR = 5.20; PD, OR = 4.01). Restless legs were more likely to be reported in all KD groups, with those on HD (OR = 6.00) and PD (OR = 9.01) more likely to report this as a symptom. The need to urinate more often was 4 to 7 times more likely to be reported in those with KD (Stage 1–2, 3, 4, 5ND, KTR). The odds of reported feeling cold as a symptom were increased across all KD stages, although were most likely found in those with Stage 5ND (OR = 8.76) and PD (OR = 13.75).

### Differences between sex: males vs. females

Figure 1 shows the differences in the number of symptoms stratified by sex, whilst Fig. 2 showed differences in the frequency and ranking of symptoms between sexes. Females had a greater number of total symptoms in the non-KD group (*P* < .001), Stage 3 (*P* < .001), Stage 5ND (*P* < .019), HD (*P* = .001) and KTRs (*P* < .001). No difference was seen for Stage 1–2 (*P* = .622), Stage 4 (*P* = .570) or PD (*P* = .966). Logistic regression models showed that being female independently increased the likelihood of reporting 11 of the 13 symptoms: sleep disturbance/insomnia (OR = .555, *P* < .001); loss of appetite (OR = .532, *P* < .001); feeling tired (OR = .481, *P* < .001); pain in bones/joints (OR = .634, *P* < .001); poor concentration/mental alertness (OR = .709, *P* = .001); loss of libido (OR = .622, *P* < .001); loss of muscle strength/power (OR = .690, *P* = .001); shortness of breath (OR = .741, *P* = .004); muscle stiffness/cramp (OR = .678, *P* < .001); restless legs (OR = .707, *P* = .001); and feeling cold (OR = .511, *P* < .001). But not itching (OR = 1.134, *P* = .222) or need to urinate more often (OR = .854, *P* = .123).

## Discussion

The present study describes how the symptoms experienced by those with KD change with advancing disease stage. The main findings can be summarised as follows: 1) symptom number increases as KD severity worsens; 2) those with early stage KD (i.e., Stages 1–2) have a comparable number of symptoms to those without KD; 3) apart from those receiving PD, the most frequently reported symptom across every group, including the non-KD group, was ‘feeling tired’; and 4) symptom burden reporting was increased in females. To our knowledge, this study is the first to present data comparing symptoms between individuals living with and without KD. Our data suggest that the most common symptoms (e.g., feeling tired, sleep disturbance, pain in bones/joints) experienced by those with KD are not necessary dependent on having KD or disease status. Nonetheless, a greater proportion of individuals living with KD report these symptoms.

As KD progresses, it does so under an array of increasing complex physiological, psychological, and treatment burden. Therefore, it is unsurprising that the number of symptoms increases as KD worsens in our current context. We found that those with mild to moderate KD (i.e., Stage 1–3) experienced ~ 4–6 symptoms. We saw no difference in the number of symptoms experienced by those between Stage 4, 5 and those on HD. Although stratifying by sex showed female patients in Stage 5ND reported the highest number of symptoms out of all groups. These findings support previous research [2, 18] where burden of symptoms is comparable in patients with end-stage KD (ESKD) and advanced KD (Stage 4–5). As a group, PD patients reported the greatest number of symptoms. Having a transplant reduced the number of symptoms experienced, although symptom numbers reported in female patients remained comparable to those on dialysis treatment. Comparing symptoms across studies is difficult due the heterogeneous use of symptom assessment tools. Previous research by our group [15], using an early version of the Leicester KSQ, found a median number of 6 symptoms in a sample of 272 patients Stage 1–5. A review of symptom burden by Almutary et al. [3] found that, across 19 included studies (and 1174 patients), the number ranged between 6 and 20 symptoms. As stated previously, the mechanisms behind why symptoms increase with disease progression is multifaceted. It is likely that uraemia and complications associated with advanced stages of KD (particularly in Stage 5, with and without dialysis) such as anaemia, metabolic derangements and fluid retention may contribute [11].

Apart from those receiving PD, the most frequently reported symptom across every group, including the non-KD group, was ‘feeling tired’, between 66 and 86% of all participants reported it as a symptom at least once a week or more. Although it was the most common symptom in the non-KD group, ‘feeling tired’ was two to five times more likely to be reported in those with KD, suggesting that having KD increases the occurrence of this symptom. Feeling tired is often used interchangeably with the term ‘fatigue’ in research and this symptom, and its variations, is often reported as the most common symptom in KD [6, 9, 10, 15, 19]. Almutary et al. [3] suggested that ‘fatigue or lack of energy’ has a weighted prevalence of 81% (49–100%) across all stages. Mechanisms of fatigue in KD are largely understudied. However, inflammation and the autonomic nervous system may be associated with fatigue in KD [20]. Other chronic illnesses (e.g., COPD, cancer) are associated with underlying biological changes (e.g., hypothalamic–pituitary–adrenal axis dysregulation) that may also contribute to fatigue reported [21]. The early assessment and management of fatigue in KD is likely to indirectly improve other symptoms, such as sleep disturbance or depression [22]. As such, greater attention to fatigue assessment is warranted and development of interventions that focus on fatigue management are required.

The need to urinate more often – also known as polyuria - was the second most common symptom in those with Stage 1 to 4, and it was the third was common symptom in those in Stage 5ND and in KTRs. Those with non-dialysis dependent KD were four to seven times more likely to report it as a symptom than those without KD. Unsurprisingly, polyuria was the least reported symptom in those on dialysis (HD and PD). Polyuria is often not included in kidney symptom questionnaires leading to a paucity of data on its prevalence [3]. Indeed, this symptom was added to the Leicester KSQ following feedback from patients, suggesting its importance to patients but under-recognition by researchers. An increased need to urinate is a symptom often attributed to having KD and it has been empirically shown that the capability to concentrate urine is impaired as kidney function deteriorates, especially at night (i.e., polynocturia) [23]. Polynocturia has been associated with non-dipper type blood pressure and is related to osmotic diuresis mainly by natriuresis rather than to water diuresis or urea excretion [23].

It is estimated that 10–20% of the general adult population suffers from some form of chronic pain [24]. Our findings showed that ‘pain in bones/joints’ was the third most frequently reported symptom in those without KD but also in those in Stage 3 and 4. It also was highly prevalent in those in Stage 1–2 and KTRs. In a community based cross-sectional study of randomly selected 1174 KD patients in Sri Lanka, ‘bone/joint pain’ was the most experienced symptom (87%; 95%CI 86–90) in a sample largely consistent of individuals in Stage 4 [11]. In Almutary et al. [3], bone or joint pain had a weighted prevalence of 24–58% across all stages, and similar findings were reported elsewhere [9]. This supports our current findings where ‘pain in bones/joints’ was reported in 39–68% of all participants across all groups. Analysis showed the odds of reporting pain in bones/joints was approximately twice as likely in those with Stage 4, HD, and KTRs compared to those without KD. Chronic pain is a well-known problem in those with advanced KD affecting around 50% of patients with ESKD [25] and pain in bones and joints is a symptom of renal osteodystrophy which often presents in those receiving long-term dialysis treatment. More general chronic musculoskeletal pain is a common symptom in KD [10, 18, 26] – including those with a transplant [27] - and may be attributed to different processes, unrelated to chronic uraemia, such as bone and mineral disorders, neuritis, or inflammatory or degenerative osteoarthritis.

In the general population, women are more likely to report symptoms than men although less is known about sex differences in the manifestation of KD. In our study, females had a greater number of total symptoms in the non-KD group, Stage 3, Stage 5ND, HD, and KTRs, and being female independently increased the likelihood of reporting 11 of the 13 symptoms. This finding is consistent with virtually all other research into KD symptom burden [11, 15, 28, 29]. Results from the EQUAL study in almost 1500 elderly European KD patients with advanced disease progression showed significant differences in the type of uraemic symptoms between women and men. Specifically, bone or joint pain, leg swelling, trouble staying asleep and shortness of breath were predominantly reported by women, while men more often reported difficulty in becoming sexually aroused. Sex differences in symptom experienced were not necessarily due to clinical status [6]. Other studies have suggested that male patients in Western countries are less willing to report symptoms [28, 29]. Although the self-reported presence of uraemic symptoms may reflect a ‘subjective’ measure of the severity of disease, the different prevalence of patient-reported symptom burden observed in men and women implies that disease manifestation, or at least disease perception, is worse in women than in men [6]. It is important to note that more symptoms are likely to be experienced in people with concomitant comorbid conditions associated with KD [3]. In our analysis, we adjusted for age, sex, ethnicity, and the number of comorbidities. This suggests that the increased symptom burden experienced across those living with KD is independent of multimorbidity.

The main strength of this study is that we were able to investigate a large cohort of individuals across the spectrum of KD and from different sites across the UK. We were also able to recruit a cohort of participants without KD to form a unique comparative group. Limitations include the cross-sectional design and limited clinical data we were able to collect for the non-KD group that restricts our ability to comprehensively detail its generalisability and representativeness. Furthermore, given the nature of KD, some participants recruited as part of the non-KD group may have some degree of KD (e.g., proteinuria) which they may not be aware of and this may distort interpretation of data in this group. However, given that these participants are unware of any such condition, it is likely that the majority do not have a KD diagnosis. Our samples were opportunistically recruited as part of an exploratory study and as such no formal sample size was calculated. However, our cohort size of over 2400 participants is likely adequate enough to draw conclusions from our data. We used the Leicester KSQ to assess symptom burden in our group. This questionnaire has been developed and validated by for use in those with non-dialysis KD [14], although its accuracy in those with end-stage KD and in KTRs remains unknown. The considerable variation in instruments, and the reporting of symptom experience (e.g., number of symptoms, types of symptoms included (i.e., physical, mental and emotional)), time period, symptom dimensions (prevalence, frequency, severity), and using different assessment scales (e.g., Likert scales), used across the literature makes interpreting our data challenging and further limits the understanding of symptom burden in different stages of KD. It is also important to note that those with early KD in our cohort were younger than those with advanced KD, and this may influence the interpretation of some symptoms where age could impact prevalence (e.g., pain); age was used as a co-variant in any statistical adjustments where possible.

## Conclusion

Empirical research spanning over a decade has showed those with KD suffer from high symptom burden. Nonetheless, these symptoms and their impact are not being appropriately managed or alleviated. Our findings have important implications for patients with KD. We have shown that high symptom burden is prevalent across the spectrum of disease, and present novel data on symptoms experienced in those without KD. Symptom burden should be assessed, either via self-report or clinician enquiry, to ensure adequate intervention, if appropriate, is implemented. Furthermore, additional research is required to develop effective assessment and management strategies for various symptoms in KD. Multidisciplinary approaches aimed at treating symptoms are needed to help reduce the numerous symptoms experienced by patients. The symptoms requiring the most immediate attention, given their high prevalence and negative impact on HRQoL, may include pain and fatigue patients and should be prioritised as an important part of disease management.

## Supplementary Information

**Additional file 1: Supplementary Material 1:** Missing symptom data. **Supplementary Material 2**: Frequency of symptoms reported across groups. **Supplementary Material 3**: Breakdown of responses in top three symptoms across groups. **Supplementary Material 4**: Odds ratio of having a symptom present across groups.

## Data Availability

The datasets used and/or analysed during the current study are available from the corresponding author on reasonable request.

## Abbreviations

95%CI: 95% confidence interval
ESKD: End-stage kidney disease
HD: Haemodialysis
HRQoL: Health related quality of life
KD: Kidney disease
KSQ: Leicester Kidney Symptom Questionnaire
KTR: Kidney transplant recipient
ND: Non-dialysis
OR: Odds Ratio
PD: Peritoneal dialysis
