# Supplementary material for: Differences in physical symptoms between those with and without kidney disease: a comparative study across disease stages in a UK population
Source: BMC Nephrol. 2021 Apr 22;22:147. doi: 10.1186/s12882-021-02355-5 (PMC8063370; doi:10.1186/s12882-021-02355-5)
Supplement: Supplementary file 1 — Additional file 1: Supplementary Material 1: Missing symptom data. Supplementary Material 2: Frequency of symptoms reported across groups. Supplementary Material 3: Breakdown of responses in top three symptoms across groups. Supplementary Material 4: Odds ratio of having a symptom present across groups. [file 12882_2021_2355_MOESM1_ESM.docx]

**Differences in physical symptoms between those with and without kidney disease: a comparative study across disease stages in a UK population**

Thomas J. Wilkinson, Daniel GD. Nixon, Jared Palmer, Courtney J. Lightfoot, Alice C. Smith

**Supplementary Material 1: Missing symptom data**

| **Symptom** | **Non-KD reference** | **Non-dialysis kidney disease** | | | | **HD** | **PD** | **KTRs** |
| --- | --- | --- | --- | --- | --- | --- | --- | --- |
|  |  | **Stage 1-2** | **Stage 3** | **Stage 4** | **Stage 5ND** |  |  |  |
|  | *N=853* | *N=72* | *N=182* | *N=194* | *N=82* | *N=630* | *N=28* | *N=230* |
| Itching (%) | 4 | 8 | 7 | 9 | 9 | 5 | 7 | 8 |
| Sleep disturbance/insomnia (%) | 1 | 4 | 2 | 5 | 8 | 2 | 0 | 4 |
| Loss of appetite (%) | 2 | 4 | 8 | 9 | 8 | 4 | 0 | 5 |
| Feeling tired (%) | 2 | 6 | 3 | 5 | 10 | 1 | 0 | 3 |
| Pain in bones/joints (%) | 2 | 8 | 8 | 5 | 4 | 3 | 0 | 7 |
| Poor concentration/mental alertness (%) | 2 | 8 | 4 | 8 | 10 | 4 | 0 | 4 |
| Loss of libido (%) | 7 | 10 | 23 | 26 | 26 | 16 | 7 | 12 |
| Loss of muscle strength/power (%) | 3 | 10 | 10 | 10 | 13 | 3 | 4 | 5 |
| Shortness of breath (%) | 2 | 8 | 7 | 6 | 10 | 2 | 0 | 5 |
| Cramp/muscle stiffness (%) | 2 | 10 | 6 | 7 | 10 | 4 | 0 | 4 |
| Restless legs (%) | 2 | 10 | 7 | 7 | 8 | 4 | 4 | 5 |
| The need to urinate more often (night and/or day) (%) | 4 | 11 | 8 | 14 | 12 | 8 | 4 | 9 |
| Feeling cold (%) | 2 | 10 | 3 | 6 | 8 | 1 | 0 | 4 |
| *Percentages shown as valid percent (i.e. excluding missing data). KD = Kidney disease; ND = Non-dialysis; HD = Haemodialysis; PD = Peritoneal dialysis; KTRs = Kidney transplant recipients. The range was 0 to 26%. Excluding loss of libido, the range was 0 to 14%.* | | | | | | | | |

**Supplementary Material 2: Frequency of symptoms reported across groups**

| **Symptom** | **Non-KD reference** | **Non-dialysis kidney disease** | | | | **HD** | **PD** | **KTRs** |
| --- | --- | --- | --- | --- | --- | --- | --- | --- |
|  |  | **Stage 1-2** | **Stage 3** | **Stage 4** | **Stage 5ND** |  |  |  |
|  | *N=853* | *N=72* | *N=182* | *N=194* | *N=82* | *N=630* | *N=28* | *N=230* |
| Itching (n, %) | 141 (17%) | 23 (35%) | 58 (34%) | 70 (40%) | 42 (51%) | 300 (50%) | 16 (62%) | 61 (29%) |
| Sleep disturbance/insomnia (n, %) | 365 (43%) | 43 (62%) | 101 (57%) | 112 (61%) | 50 (60%) | 375 (61%) | 15 (54%) | 132 (60%) |
| Loss of appetite (n, %) | 81 (10%) | 12 (17%) | 27 (16%) | 43 (24%) | 30 (36%) | 244 (40%) | 15 (54%) | 65 (30%) |
| Feeling tired (n, %) | 549 (66%) | 54 (79%) | 137 (77%) | 146 (79%) | 69 (85%) | 535 (86%) | 22 (79%) | 175 (79%) |
| Pain in bones/joints (n, %) | 327 (39%) | 30 (46%) | 98 (58%) | 125 (68%) | 54 (63%) | 363 (59%) | 16 (57%) | 119 (55%) |
| Poor concentration/mental alertness (n, %) | 221 (27%) | 24 (36%) | 52 (30%) | 68 (38%) | 33 (41%) | 234 (39%) | 12 (43%) | 95 (43%) |
| Loss of libido | 167 (21%) | 13 (18%) | 54 (30%) | 81 (56%) | 30 (45%) | 180 (34%) | 14 (54%) | 84 (42%) |
| Loss of muscle strength/power (n, %) | 114 (14%) | 21 (32%) | 76 (46%) | 98 (56%) | 46 (59%) | 417 (68%) | 16 (59%) | 116 (53%) |
| Shortness of breath (n, %) | 101 (12%) | 18 (27%) | 77 (45%) | 103 (56%) | 44 (54%) | 330 (54%) | 17 (61%) | 91 (42%) |
| Cramp/muscle stiffness (n, %) | 189 (23%) | 24 (37%) | 80 (47%) | 92 (51%) | 52 (64%) | 385 (63%) | 16 (57%) | 110 (50%) |
| Restless legs (n, %) | 113 (14%) | 19 (29%) | 56 (33%) | 69 (38%) | 29 (35%) | 354 (59%) | 15 (56%) | 70 (32%) |
| The need to urinate more often (night and/or day) (n, %) | 199 (24%) | 41 (64%) | 108 (64%) | 122 (74%) | 51 (65%) | 189 (32%) | 10 (37%) | 122 (58%) |
| Feeling cold (n, %) | 213 (25%) | 30 (46%) | 94 (53%) | 124 (68%) | 61 (74%) | 383 (62%) | 23 (82%) | 111 (51%) |
| No. of symptoms/13 | 3.3 (2.7) | 5.0 (3.1) | 5.6 (3.2) | 6.5 (3.3) | 6.6 (3.5) | 6.6 (3.1) | 7.4 (2.8) | 6.0 (3.5) |
| *Percentages shown as valid percent (i.e. excluding missing data). Present symptom defined as being present at least once a week or more; absence of symptom was defined as either never or <1 week. KD = Kidney disease; ND = Non-dialysis; HD = Haemodialysis; PD = Peritoneal dialysis; KTRs = Kidney transplant recipients.* | | | | | | | | |

**Supplementary Material 3: Breakdown of responses in top three symptoms across groups**

| **Symptom** | **Non-KD reference** | **Non-dialysis kidney disease** | | | | **HD** | **PD** | **KTRs** |
| --- | --- | --- | --- | --- | --- | --- | --- | --- |
|  |  | **Stage 1-2** | **Stage 3** | **Stage 4** | **Stage 5ND** |  |  |  |
|  | *N=853* | *N=72* | *N=182* | *N=194* | *N=82* | *N=630* | *N=28* | *N=230* |
| **Feeling tired (n, %) – present in 76% of all participants** | | | | | | | | |
| Never | 156 (19%) | 8 (12%) | 28 (16%) | 20 (11%) | 7 (9%) | **42 (7%)**** | 1 (4%) | 26 (12%) |
| <1 week | 129 (16%) | 6 (9%) | 12 (7%) | 19 (10%) | 5 (6%) | **44 (7%)**** | 5 (18%) | 22 (10%) |
| 1-2 a week | 217 (26%) | 14 (21%) | 36 (20%) | **28 (15%)**** | 10 (12%) | **88 (14%)**** | 1 (4%) | 39 (18%) |
| Several times a week | 209 (25%) | 25 (37%) | 36 (20%) | 35 (19%) | **18 (22%)*** | **213 (34%)**** | 8 (29%) | 63 (28%) |
| Everyday | 123 (15%) | 15 (22%) | **65 (37%)**** | **83 (45%)**** | **41 (51%)**** | **234 (38%)**** | **13 (46%)**** | **73 (33%)**** |
| **Sleep disturbance/insomnia (n, %) – present in 54% of all participants** | | | | | | | | |
| Never | 310 (37%) | 18 (26%) | 55 (31%) | 58 (31%) | 25 (30%) | **158 (26%)**** | 6 (21%) | **55 (25%)**** |
| <1 week | 168 (20%) | 8 (12%) | 22 (12%) | **15 (8%)**** | 8 (10%) | **84 (14%)*** | 7 (25%) | 33 (15%) |
| 1-2 a week | 161 (19%) | 18 (26%) | 23 (13%) | 32 (17%) | 11 (13%) | 113 (18%) | 6 (21%) | 45 (21%) |
| Several times a week | 142 (17%) | 16 (23%) | 41 (23%) | 33 (18%) | 15 (18%) | 139 (23%) | 7 (25%) | 42 (19%) |
| Everyday | 62 (7%) | 9 (13%) | **37 (21%)**** | **47 (25%)**** | **24 (29%)**** | **123 (20%)**** | 2 (7%) | **45 (21%)**** |
| **Pain in bones/joints (n, %) – present in 52% of all participants** | | | | | | | | |
| Never | 366 (44%) | 21 (32%) | 53 (32%) | **45 (24%)**** | 21 (24%) | **179 (29%)**** | 9 (32%) | 74 (34%) |
| <1 week | 146 (17%) | 15 (23%) | 17 (10%) | 15 (8%) | 11 (13%) | 70 (11%) | 3 (11%) | 22 (10%) |
| 1-2 a week | 127 (15%) | 11 (17%) | 22 (13%) | 25 (14%) | 6 (7%) | 101 (17%) | 3 (11%) | 39 (18%) |
| Several times a week | 101 (12%) | 7 (11%) | 18 (11%) | 26 (14%) | 16 (19%) | 108 (18%) | 8 (29%) | 25 (12%) |
| Everyday | 99 (12%) | 12 (18%) | **58 (35%)**** | **74 (40%)**** | **32 (37%)**** | **154 (25%)**** | 5 (18%) | **55 (26%)**** |
| *Data shown as number and percentage. A present symptom was defined as being present at least once a week or more; absence of symptom was defined as either never or <1 week. The tope 3 symptoms were reported (defined as being present in over 50% of all participants). For males, loss of libido also included presence of erectile dysfunction. ND = Non-dialysis; HD = Haemodialysis; PD = Peritoneal dialysis; KTRs = Kidney transplant recipients. Significant difference in proportion compared to non-KD group: * = P<.050, ** = P<.001* | | | | | | | | |

**Supplementary Material 4: Odds ratio of having a symptom present across groups**

| **Symptom** | **Non-dialysis kidney disease** | | | | | | | | **HD** | | **PD** | | **KTRs** | |
| --- | --- | --- | --- | --- | --- | --- | --- | --- | --- | --- | --- | --- | --- | --- |
|  | **Stage 1-2** | | **Stage 3** | | **Stage 4** | | **Stage 5ND** | |  |  |  |  |  |  |
|  | **OR (95%)** | **P** | **OR (95%)** | **P** | **OR (95%)** | **P** | **OR (95%)** | **P** | **OR (95%)** | **P** | **OR (95%)** | **P** | **OR (95%)** | **P** |
| Itching | 2.28 (1.31 to 3.97) | **.004*** | 2.79 (1.86 to 4.21) | **<.001*** | 3.40 (2.22 to 5.19) | **<.001*** | 5.25 (3.12 to 8.81) | **<.001*** | 5.51 (4.09 to 7.41) | **<.001*** | 7.62 (3.31 to 17.56) | **<.001*** | 1.75 (1.19 to 2.55) | **.004*** |
| Sleep disturbance/insomnia | 2.40 (1.42 to 4.06) | **.001*** | 1.58 (1.09 to 2.28) | **.016*** | 1.68 (1.14 to 2.49) | **.009*** | 1.77 (1.07 to 2.92) | **.027*** | 2.19 (1.69 to 2.85) | **<.001*** | 1.49 (0.67 to 3.30) | .329 | 1.84 (1.32 to 2.57) | **<.001*** |
| Loss of appetite | 2.13 (1.07 to 4.22) | **.031*** | 2.36 (1.39 to 4.00) | **.001*** | 4.00 (2.43 to 6.58) | **<.001*** | 6.74 (3.82 to 11.89) | **<.001*** | 8.80 (6.19 to 12.51) | **<.001*** | 13.60 (5.9 to 31.50) | **<.001*** | 4.55 (3.01 to 6.89) | **<.001*** |
| Feeling tired | 2.01 (1.07 to 3.76) | .**029*** | 1.97 (1.27 to 3.06) | .**002*** | 2.11 (1.32 to 3.38) | .**002*** | 3.42 (1.74 to 6.75) | **<.001*** | 4.64 (3.32 to 6.47) | **<.001*** | 2.02 (0.78 to 5.28) | .150 | 1.93 (1.32 to 2.85) | .**001*** |
| Pain in bones/joints | 1.26 (0.74 to 2.13) | .339 | 1.42 (0.97 to 2.08) | .073 | 1.71 (1.14 to 2.55) | **.009*** | 1.50 (.91 to 2.48) | .113 | 1.72 (1.33 to 2.24) | **<.001*** | 1.30 (.58 to 2.92) | .522 | 1.47 (1.05 to 2.05) | .**026*** |
| Poor concentration/mental alertness | 1.43 (0.83 to 2.48) | .199 | 1,31 (.87 to 1.98) | .193 | 2.08 (1.36 to 3.16) | .**001*** | 2.02 (1.19 to 3.44) | .**009*** | 2.29 (1.72 to 3.03) | **<.001*** | 1.78 (.78 to 4.06) | .171 | 1.94 (1.38 to 2.73) | **<.001*** |
| Loss of libido | 1.00 (.52 to 1.94) | .991 | 1.59 (1.04 to 2.44) | **.032*** | 2.61 (1.69 to 4.05) | **<.001*** | 1.85 (1.06 to 3.23) | **.030*** | 1.58 (1.16 to 2.13) | **.003*** | 3.43 (1.46 to 8.08) | .**005*** | 2.43 (1.68 to 3.53) | **<.001*** |
| Loss of muscle strength/power | 3.15 (1.77 to 5.62) | **<.001*** | 3.26 (2.16 to 4.90) | **<.001*** | 3.81 (2.49 to 5.82) | **<.001*** | 4.93 (2.89 to 8.42) | **<.001*** | 9.66 (7.12 to 13.01) | **<.001*** | 6.19 (2.70 to 14.24) | **<.001*** | 5.47 (3.81 to 7.87) | **<.001*** |
| Shortness of breath | 2.39 (1.30 to 4.37) | **.005*** | 4.65 (3.08 to 7.03) | **<.001*** | 6.14 (4.02 to 9.40) | **<.001*** | 6.09 (3.59 to 10.31) | **<.001*** | 7.78 (5.68 to 10.66) | **<.001*** | 8.39 (3.79 to 19.11) | **<.001*** | 4.12 (2.84 to 5.97) | **<.001*** |
| Cramp/muscle stiffness | 2.04 (1.18 to 3.50) | **.010*** | 2.48 (1.69 to 3.63) | **<.001*** | 2.59 (1.74 to 3.87) | **<.001*** | 4.81 (2.85 to 8.12) | **<.001*** | 5.20 (3.94 to 6.86) | **<.001*** | 4.01 (1.79 to 8.99) | .**001*** | 2.90 (2.07 to 4.08) | **<.001*** |
| Restless legs | 2.54 (1.41 to 4.55) | **.002*** | 3.76 (2.46 to 5.72) | **<.001*** | 4.80 (3.11 to 7.40) | **<.001*** | 3.86 (2.25 to 6.65) | **<.001*** | 6.00 (4.38 to 8.22) | **<.001*** | 9.01 (3.92 to 10.70) | **<.001*** | 2.92 (2.00 to 4.26) | **<.001*** |
| The need to urinate more often (night and/or day) | 5.36 (3.11 to 9.25) | **<.001*** | 4.75 (3.21 to 7.03) | **<.001*** | 7.16 (4.60 to 11.14) | **<.001*** | 4.78 (2.83 to 8.07) | **<.001*** | 1.24 (.93 to 1.65) | .138 | 1.64 (.73 to 3.70) | .231 | 4.02 (2.85 to 5.68) | **<.001*** |
| Feeling cold | 2.83 (1.66 to 4.84) | **<.001*** | 3.51 (2.39 to 5.14) | **<.001*** | 5.93 (3.92 to 8.98) | **<.001*** | 8.76 (5.01 to 15.36) | **<.001*** | 5.67 (4.27 to 7.52) | **<.001*** | 13.75 (5.02 to 37.71) | **<.001*** | 3.03 (2.15 to 4.27) | **<.001*** |
| *Data shown as Odds Ratio with 95% confidence intervals. The dotted line represents an Odds Ratio of 1 (non-kidney disease reference group). An Odds Ration greater than 1 indicated an increased likelihood of having this symptom. Closed black dots indicate a significant Odds Ratio; larger open square indicates no statistically significant difference to reference group. A present symptom was defined as being present at least once a week or more; absence of symptom was defined as either never or <1 week. For males, loss of libido also included presence of erectile dysfunction. Tested using multinominal logistic regression modelling (adjusted for age, sex, ethnicity, and no. of comorbidities). ND = Non-dialysis; HD = Haemodialysis; PD = Peritoneal dialysis; KTRs = Kidney transplant recipients* | | | | | | | | | | | | | | |
